# Sudden Death from Primary Cerebral Melanoma: Clinical Signs and Pathological Observations

**DOI:** 10.3390/healthcare9030341

**Published:** 2021-03-17

**Authors:** Alfonso Maiellaro, Antonio Perna, Pasquale Giugliano, Massimiliano Esposito, Giuseppe Vacchiano

**Affiliations:** 1 Legal Medicine Department, A. Cardarelli Hospital, 80131 Naples, Italy; alfonso.maiellaro@aocardarelli.it; 2 Pathology Unit, Mauro Scarlato Hospital, 84018 Scafati, Italy; antoniopernadoc@libero.it; 3 AORN Sant’Anna e San Sebastiano di Caserta, 81100 San Sebastiano, Italy; dottgiugliano@tiscali.it; 4 Legal Medicine, Department of Medical, Surgical and Advanced Technologies, “G.F. Ingrassia”, University of Catania, 95123 Catania, Italy; 5 Department of Law, University of Sannio, 82100 Benevento, Italy

**Keywords:** primary cerebral melanoma, sudden death, clinical signs, pathological observations

## Abstract

Primary cerebral tumors rarely provoke sudden death. The incidence is often underestimated with reported frequencies in the range of 0.02 to 2.1% in medicolegal autopsy series. Furthermore, primary cerebral melanoma is an uncommon neoplasm. It represents approximately 1% of all melanoma cases and 0.07% of all brain tumors. This neoplasm is very aggressive, and its annual incidence is about 1 in 10 million people. In the present study, a 20-year-old male was admitted to hospital with vomiting, headache, paresthesia and aggressive behavior. A computed tomography (CT) scan of the head was performed showing a hyperdense nodule in the right parietal lobe with inflammation of the Silvian fissure. A complete autopsy was performed 48 h after death. A blackish material was displayed on the skull base, and posterior fossa. Microscopic examination diagnosed primary brain melanoma. A systematic review of the literature was also performed where no previous analogous cases were found. The forensic pathologist rarely encounters primary cerebral melanoma, and for these reasons, it seemed appropriate to describe this case as presenting aspecific clinical symptoms and leading to sudden unexpected death. Histopathological observations are reported and discussed to explain this surprising sudden death caused by a primary cerebral melanoma.

## 1. Introduction

Sudden unexpected death due to a primary nervous neoplasm is not frequent in medico-legal activity [1] because the incidence of undiagnosed primary central nervous system tumors has decreased with the emergence of advanced neuroimaging and other improved diagnostic techniques. Occasionally, the forensic pathologist can encounter a cerebral neoplasm that had been undiagnosed or not suspected prior to death. Eberhart et al. [2] observed 11 cases of primary central nervous system tumors resulting in sudden death over a period of 20 years (1980 to 1999). Glioblastomas [3,4,5,6], oligodendragliomas [7], astrocitomas [8], ependymomas [9], extramedullary plasmacytomas [10], colloid cysts of third ventricle [11,12] and meningiomas [13], are reported in the literature to cause sudden death with vague or short-term symptoms and limited healthcare access. Current studies of sudden death from brain tumors concern glioblastoma multiforme or astrocytomas. In the present study, a systematic review of sudden death and brain tumors from 1980 to 2021 was conducted, and it was concluded that although articles had been analyzed over a period of 40 years, there are still few articles today that report this type of death. We found only one case of sudden death associated with melanoma brain metastases [14] and it seemed appropriate to describe the histopathological manifestations of this surprising case of sudden death due to primary cerebral melanoma. Glitza [15], in an autopsy series, identified up 80% of patients with melanoma with metastatic involvement of the central nervous system (CNS); however, while the cerebral metastases of melanoma are often observed, primary cerebral melanoma is very rare. Several histogenetic theories on the origin of meningeal melanocytes have been put forward [16]. It has been postulated that during embryogenesis, leptomeningeal melanocytes coming from the multipotential cells of the neural crest can develop into mesodermal and neural elements. Primary cerebral melanomas develop once melanocytes become neoplastic.

The aim of this study was to present a case report and a systematic review of this rare pathology, in order to improve the overall knowledge of forensic pathologists on sudden death from brain tumors, especially in the case of cerebral melanoma. A complete autopsy, comparing radiological findings and histological presentation, could provide a comprehensive overview of such sudden deaths.

## 2. Materials and Methods

### 2.1. Case Description

**Figure 1:**
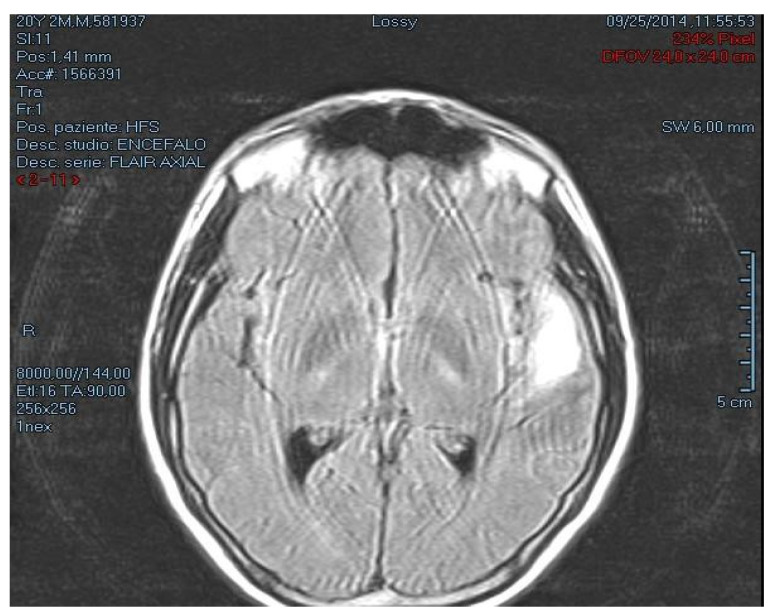
Pre-mortem CT scan of the brain. The right parietal lobe showed inflammatory processes with a hyperdense nodule.

A 20-year-old male was admitted to hospital with vomiting and headache; he was a cannabis user and cigarette smoker (40 cigarettes a day). He referred paresthesia in the upper limbs, psychomotor agitation and aggressive behavior. The neurological examination pointed out a palpebral ptosis and a right eye ophthalmoplegia including paralysis of the left facial nerve. A CT scan of the brain was performed and showed a hyperdense nodule in the right parietal lobe and along the Sylvian fissure, the leptomeningeal spaces were described as affected by an inflammatory process (Figure 1). Fifteen minutes after the CT scan, he died. The study of the medical record and the sudden onset of symptoms defined death as “sudden”. The accuracy of the autopsy examination, the histological investigation (H&E and immunohistochemistry), attributed his death to a rare form of primary cerebral melanoma.

No ethical committee was required. Written informed consent was obtained from his relatives.

### 2.2. Autopsy Findings

**Figure 2:**
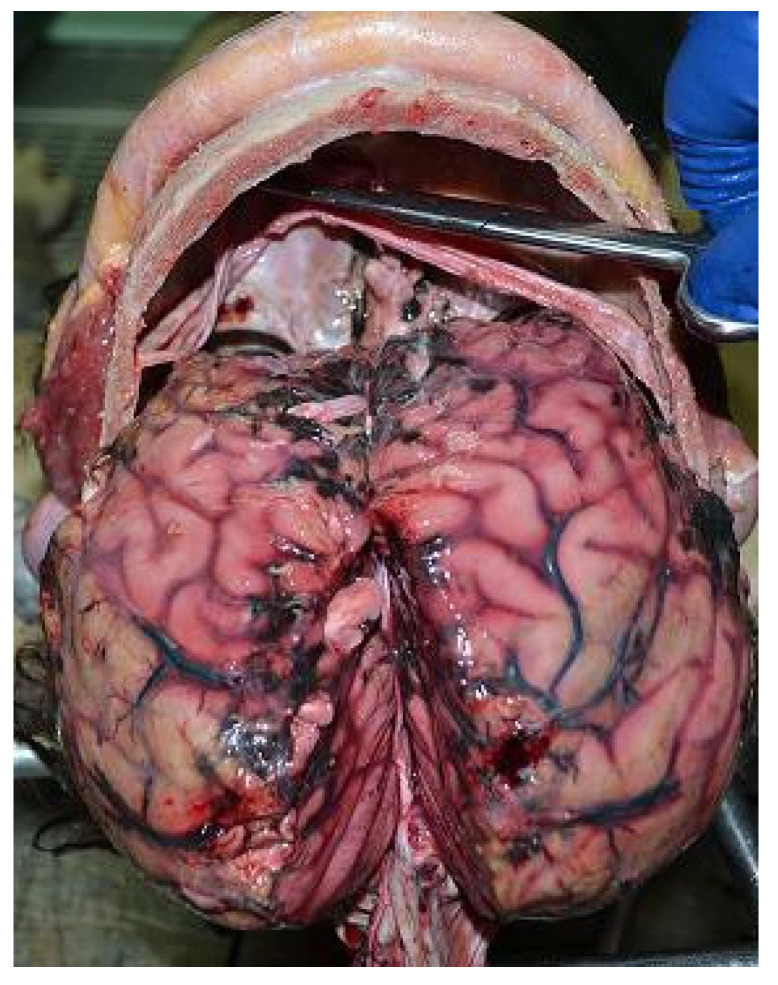
Brain examination. In the temporal and parietal lobes, some nodular blackish rounded soft formations with blood were observed.

**Figure 3:**
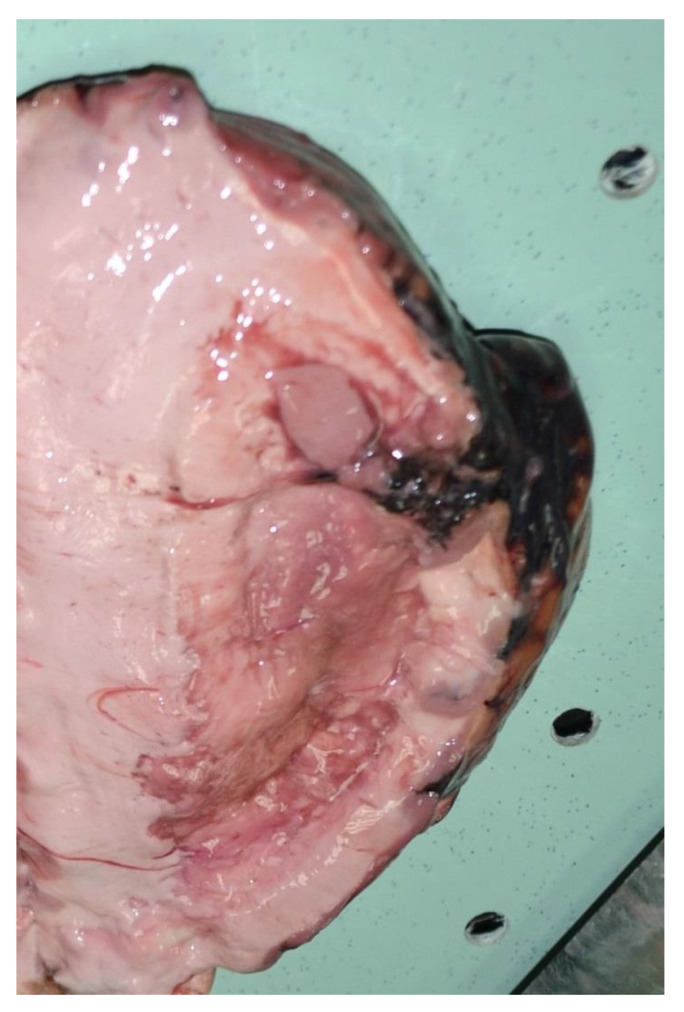
Posterior face of the brain revealed stem striae of blackish material that invaded the nervous tissue.

**Figure 4:**
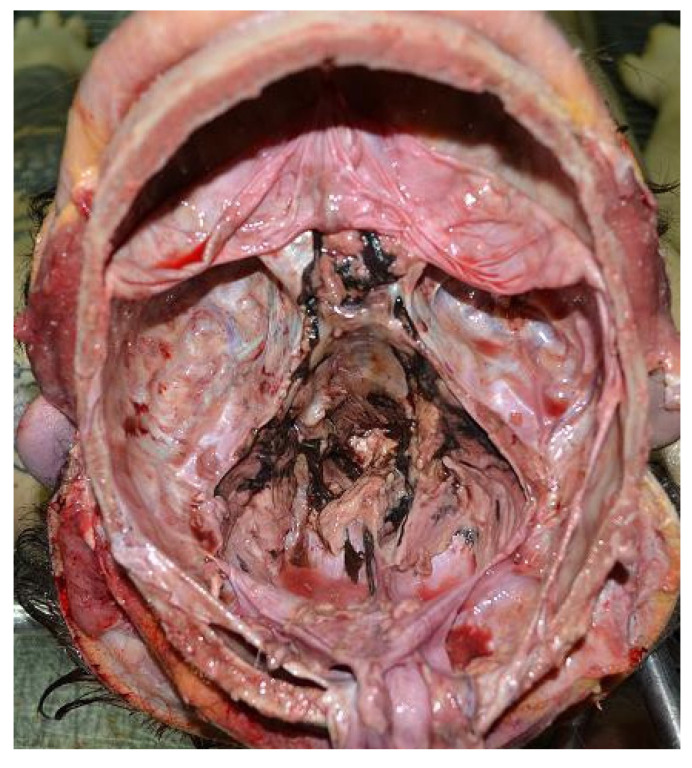
Skull base-posterior fossa and venous sinus examination showed a blackish material.

A complete autopsy was performed 48 h after death. External examination revealed abrasions on the hands and the upper limbs. The autopsy revealed the dura was full of clots. The brain weighed 1550 g and was diffusely swollen. In the subarachnoid space of the fronto-parietal lobes and along the interhemispheric cleavage of the parietal left lobe, some nodular blackish rounded soft formations with blood were observed. Moreover, in the temporal and parietal lobes, similar formations were present (Figure 2). A soft and blackish mass (4.5 × 7 cm) enveloped the brain stem and was spreading to the inferior temporal poles and to the cerebellar lobes. There was no herniation of the temporal lobe, unci or cerebellar tonsils. The section of the brain revealed on edematous soft parenchyma. Blackish soft material present in subarachnoid space of the fronto-parietal right lobe invaded the cerebral cortex. The brain ventricles were filled with blood. From the dorsal face of the brain stem, striae of blackish material invaded the nervous tissue (Figure 3). Blackish material was also present along the venous sinus of the skull base and in the posterior fossa (Figure 4). The internal examination of the other organs was unmarkable.

### 2.3. Histological Analysis

**Figure 5:**
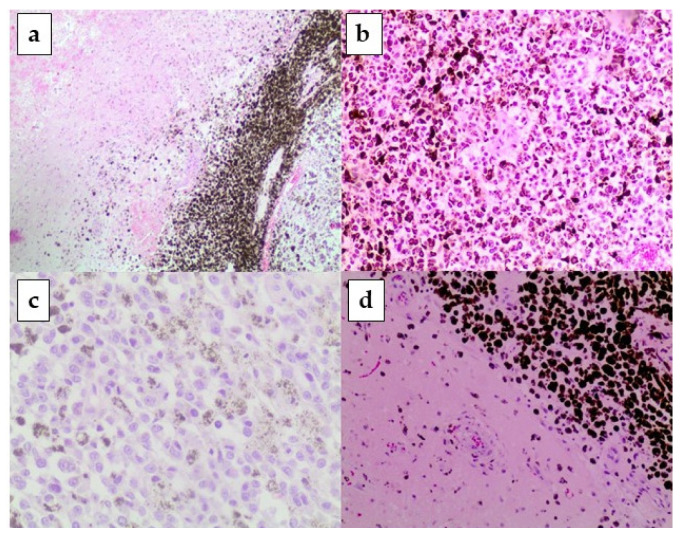
Histological findings. (**a**) neoplastic voluminous cells with melanin in the cerebral tissue (E.E. 60×); (**b**) neoplastic cell population: numerous, voluminous melanoblasts with marked nuclear pleiomorphism; (**c**) neoplastic cells with melanic pigment antimelanoma-antibody (HMB45 + 200×); (**d**) neoplastic cell population near a small cerebral artery (E.E. 200×).

During the autopsy, brain samples were collected that were fixed in 10% buffered formalin. After washing, the water was removed, and they were embedded in paraffin. The obtained blocks were cut (4 µm thickness), using a microtome (Dako, Glostrup, Denmark), sectioned, and stored at room temperature. Sections were stained with Hematoxylin and Eosin (H&E) and Von Gieson’s method. The immunohistochemical analysis of the samples was performed through antimelanoma-antibody (HMB45+), Melan A-antibody, CD3-antibody, CD20+-antibody, PenCK-antibody, Ki67+, as suggested by Tosaka et al. [17] for the diagnosis of primary leptomeningeal melanoma. Sections were observed using a Zeiss Axioplan light microscope (Carl Zeiss, Oberkochen, Germany). Subsequently, images were obtained using a Zeiss AxioCam MRc5 digital camera (Carl Zeiss, Oberkochen, Germany). In the present study, the microscopic examination of the brain showed, on the right parietal lobe and in brain-stem tissues, many atypical and pleomorphic cells with voluminous discolored nuclei and melanin-pigment in the cytoplasm. These cells were arranged like a “cordon” or irregularly like a “vortex”. Moreover, a vascular proliferation and spotty hemorrhages were also observed. In the cerebral cortex tissues, edema and ischemic dark neurons, and near them, a lymphocytic and macrophage reaction were seen. The leptomeningeal arteriolar vessels showed clots, partially reorganized. Around them, we observed many pleomorphic and atypical cells with melanin pigment in the cytoplasm (Figure 5).

## 3. Systematic Review

A systematic review was conducted according to the PRISMA guidelines [18].

Pubmed and Google Scholar were used as search engines from 1 January 1980 to 1 February 2021 to evaluate the association between sudden death and brain tumor. meSH was used for the following words: (sudden death) AND (primary cerebral tumor).

### 3.1. Inclusion and Exclusion Criteria

The following exclusion criteria were used: (1) review, (2) articles not in English, (3) animal studies, (4) abstract, (5) editorial, (6) poster, and (7) communications at conferences. The inclusion criteria were as follows: (1) Original Article, (2) Case Report, and (3) Articles in English.

### 3.2. Quality Assessment and Data Extraction

A.M. and A.P. initially evaluated all the articles, evaluating the title, the abstract, and the whole text. Once, P.G. and M.E. reanalyzed the articles chosen independently. In cases of conflicting opinions between the articles, they were submitted to G.V.

### 3.3. Characteristics of Eligible Studies

**Figure 6:**
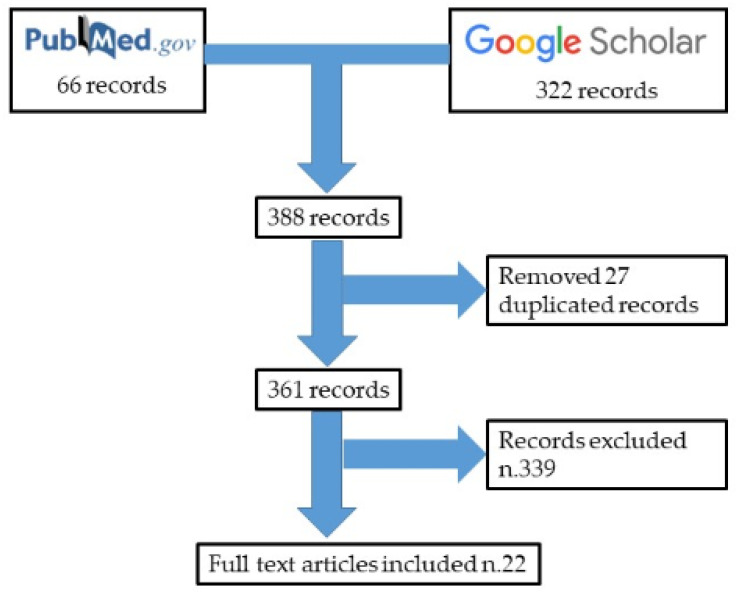
Flow diagram illustrating included and excluded studies in this systematic review.

A total of 388 articles were collected (66 from PubMed and 322 from Google Scholar). Of them, 27 duplicates were removed. A total of 339 articles did not meet the inclusion criteria. In conclusion, 22 articles were included in the present systematic review (Figure 6).

## 4. Results

Most of the studies were case reports (n. 16), some cases series (n. 4), and a few original articles (n. 2). Most of the tumors related to sudden death were matched with glioblastoma multiform or astrocytomas in the different world health organization (WHO) grades. Other tumors related to sudden death were oligodendrogliomas, gliomas, adenomas, and colloid cysts. The most frequent autopsy findings were cerebral edema, solid tumor mass, and hemorrhagic infarction of surrounding tissue. The most frequent cause of death was due to intracranial hypertension from the tumor; sometimes it was due to hemorrhage caused by the tumor (especially in cases of glioblastoma multiform); only one case was due to respiratory arrest due to the location of the tumor near the center of breathing control (Table 1).

## 5. Discussion

Sudden death due to brain tumors has a low incidence and is often underestimated [18]. This systematic review highlights that currently there is no study with a large number of cases of sudden death from brain tumors. Furthermore, most of the case reports report glioblastoma multiforme. This case report is the first to focus on sudden death from cerebral melanoma and it is important that forensic pathologists recognize this cause of sudden death.

Primary cerebral melanoma is a very uncommon neoplasm derived from melanocytes present in the leptomeninges. This pathology, first described by Virchow [32], is very rare, with an annual incidence of approximately 1 in 10 million people [33] and represents about 1% of all melanoma cases and 0.07% of all brain tumors [34]. Indeed, many studies have demonstrated that in central nervous systems (CNS) metastases of melanoma are also frequently present because the melanoma has a high risk of spreading to CNSs. The melanoma cells, in fact, share with vascular cells numerous cell surface molecules; they are highly angiogenic and possess a higher degree of “stemness” than other solid tumors [15]. According to Hayword [35], we can identify a primary cerebral melanoma when the following occur: (a) no malignant melanoma outside the CNS; (b) non-malignant neoplasm in other parts of the CNS; (c) histological validation of melanoma.

Age is also an additional factor to distinguish a primary cerebral melanoma from metastatic melanoma. Primary cerebral melanoma develops primarily in patients under 50 years of age and rarely metastasize to other organs. Metastatic melanomas develop mainly in the elderly, have a rapid clinical course and multiple intracerebral diffusion [36]. In the presented case, no other melanomas were detected at the clinical and forensic examinations of the body; a solitary blackish mass (4 × 7 cm) was observed around the brain stem. Leptomeningeal blackish soft rounded formations in the fronto-parietal lobes were observed and the patient was under 50 years old. The histological observations confirmed, with the immunohistochemical analysis, the diagnosis of melanoma. In addition to the blackish mass observed around the brain stem, we also observed in the subarachnoid space in the right parietal lobe some rounded blackish nodules invading the nervous tissue. We interpreted these modifications as the primitive cerebral melanoma located in the brain stem subarachnoid space, where we found a blackish mass at autopsy. The meningeal vessels of the left parietal lobe were thrombosed and around these vessels we observed many atypical pleomorphic cells with cytoplasmatic melanin pigment; the neoplasm had probably first developed from the leptomeningeal space around the brain stem, and after, spread to the subarachnoid space of the left parietal lobe. In fact, it should be borne in mind also that melanocytes far outnumber leptomeninges of the skull base and cervical cord [37,38] and the most common locations of primary cerebral melanoma are the anterolateral face of the spinal cord and the postero-lateral face of the brain stem [39]. In agreement with this author, primary cerebral melanoma was observed also in the posterior fossa in the cerebellopontine angle [40,41] and Arantes [42] reported 13 cases of primary malignant melanoma derived from the pineal body. Greco Castro [43] observed this neoplasm in the temporal lobe near the Sylvian fissure and Quillo-Olvera [44] reported that the primary cerebral melanoma can be found frequently in the cerebral lobe (53.1%) in the posterior fossa (17.3%) and pineal region (13.6%). We believe that primary cerebral melanoma may occur in any leptomeningeal location, although our reported case is in agreement with the observations of Troya-Castilla. The clinical manifestations are generally non-specific, related to an increased cerebral pressure, or cerebral hemorrhages, or a neurological dysfunction and the early diagnosis of this neoplasm is a very difficult challenge for clinicians [45,46,47,48]. The aggressive behavior, the vague symptoms, and the unspecific neuroimaging make the prognosis of this tumor very difficult [49]. Several studies [50,51] reported a survival of ~4 months from diagnosis of cerebral nervous system melanoma, and once the cancer spreads to the leptomeninges, the overall median survival is generally only 10 weeks [52,53]. In our case, two months after the first symptoms, the patient died. The patient had a history of drug addiction, presenting psychomotor agitation and aggressive behavior, as can be observed in many cerebral neoplasms [54,55,56,57]. Morais described a case of delirium in a patient with brain melanoma metastases [58] and other authors reported headache and diplopia in a 27-year-old man affected by primary meningeal melanocytoma in the anterior cranial fossa [59]. However, we can ascribe the first presented symptoms (headache and vomit) to increased intracranial pressure, probably due to the increased neoplastic mass in the posterior fossa and a subarachnoid hemorrhage. However, the symptoms observed before death (paralysis of the III and VIII cranial nerve) clearly showed a suffering brain stem. The involvement of this important brain structure also explains the sudden death due to an alteration of the hypothalamic cardiovascular regulatory centers. It is well-known that stimulation of the hypothalamus can lead to anatomic cardiovascular disturbances and cardiac arrest [60,61].

## 6. Conclusions

The surprising case of sudden death stresses the need for a careful analysis in all patients with psychiatric disorders or no-specific neurological signs, especially when these manifestations show-up unexpectedly in a healthy person. Despite the fact that modern genetic [62,63,64], radiological [65,66,67], and therapeutic treatments [68,69,70,71,72,73] have revolutionized the approach to primary cerebral melanoma, autopsy can still provide a useful support in determining the exact location, diffusion, and histological pattern of this neoplasm, and the cause of death. In human beings, melanocyte exists in the uvea, cerebral parenchyma, leptomeninges, and mucous membranes. Primary cerebral melanoma is rare and has an estimated incidence of 0.005 cases per 100,000 and develops in around 1% of all melanoma cases. Brain melanoma metastasis is much more common than primary brain melanoma. Primary brain melanomas originate from melanocytes of the leptomeninges and histopathologically show strongly pigmented cells with prominent nucleoli [74]. Through this systematic review, the present case is the first described in literature in which a primary brain melanoma caused a sudden death. It is, therefore, of crucial importance to report this case to the scientific community.

## Figures and Tables

**Table 1:** summarizes the details of the systematic review.

Reference	Study Design	Primary Cerebral Tumor	Autopsy Findings	Cause of Death
Eberhart, C.G. [2]	Original Article	Astrocytoma (n.2) Schwannoma (n.1) Glioblastoma multiforme (n.4) Colloid cyst (n.2) Glioma (n.1) Pituitary adenoma (n.1)	Brain edema was shown in all cases. The microscopic study diagnosed the type-specific tumor.	Death was attributed in all cases to hydrocephalus and intracranial hypertension except for one case of glioblastoma multiforme in which death was attributed to massive tumor hemorrhage.
Matschke, J. [3]	Case Series	Glioblastoma multiform (n.3)	Gross examination of the brain showed numerous cystic spaces. Microscopic examination revealed polymorphic astrocytic cells.	Death was attributed to intracranial hypertension.
Gleckman, A.M. [4]	Case Series	Ganglioma (n.1) Astrocytoma (n.1)	Brain edema was shown in all cases. The microscopic study diagnosed the type-specific tumor.	Death was attributed to hydrocephalus and intracranial hypertension.
Sutton, J.T. [5]	Case Report	Glioblastoma multiform	Gross examination of the brain showed a hemorrhagic infiltration of the right lobe equal to 7 × 5 × 5 cm. Microscopic examination revealed hemorrhagic infiltration of the cortex with tumor invasion.	Death was due to an acute hemorrhage of the tumor.
Riezzo, I. [6]	Case Series	Glioblastoma multiforme (n.3)	Macroscopic findings of the brain were characterized by diffuse hypoxia/ischemia and edema of the brain tissue with extensive hemorrhagic infiltration and necrosis confirmed also on histological examination.	Death was attributed in all cases to hydrocephalus and intracranial hypertension.
Manousaki, M. [7]	Case Report	Oligodendroglioma	Brain edema with “fried-egg” cell tumor.	Death was due to hemorrhagic leakage into the cerebrospinal fluid
Vougiouklakis, T. [8]	Case Series	Glioblastoma multiforme (n.1) Astrocytoma WHO grade III (n.1)	The examination of the brain revealed flattening of the fissures with large hemorrhagic infarction in both cases.	Death was due to massive tumor hemorrhage.
Harrison, W.T. [9]	Case Report	Anaplastic Ependymoma	After formalin fixation, the brain showed a 7 × 6 × 6 cm necrotic cavity mass of the lateral ventricle. Microscopically, the tumor has been attributed to an anaplastic ependymoma with parenchyma characterized by fibrillary processes.	Death was attributed to hydrocephalus and intracranial hypertension.
Sidlo, J. [10]	Case Report	Sellar extramedullary plasmacytoma	After brain removal, the examination of the sella turcica showed an intrasellar tumor mass of 5.5 × 5.5 × 3 cm. Histopathological examination showed mature plasma cells with eccentrically positioned round nuclei.	Death was attributed to hydrocephalus and intracranial hypertension
Aissaoui, A. [19]	Case Report	Leptomeningeal Melanocytosis	A dark brown mass was present on the basal leptomeninges in the frontal areas. The brain was edematous. Microscopic analysis revealed the dark color of the tumor due to melanin pigments.	Death was attributed to hydrocephalus and intracranial hypertension
Ozkul, A. [20]	Case Report	Leptomeningeal oligodendrogliomatosis	Macroscopic examination revealed edema of the brain. H&E examination showed an invasion of tumor at the brain, cerebellum and spinal cord by plasmacytoid cells.	Death was attributed to hydrocephalus and intracranial hypertension.
Ross, J. [21]	Case Report	Glioma	The brain was characterized by diffuse hypoxia/ischemia and edema of the brain tissue. At H&E examination, a hyper cellularity of glial tumor cells was displayed.	Death was attributed to hydrocephalus and intracranial hypertension
Havlik, D.M. [22]	Case Report	Glioma	Macroscopic examination of the brain showed swelling of hemispheres; at H&E examination, pseudo rosettes and tumor cells were seen.	Death was attributed to intracranial hypertension.
DiMaio, S.M. [23]	Original Article	Colloid cyst (n.1) Oligodendroglioma (n.2) Glioblastoma multiforme (n.2) Astrocytoma WHO grade III (n.3) Medulloblastoma (n.1) Astrocytoma WHO grade II (n.4) Sarcoma (n.1) Teratoma cyst (n.1) Meningioma (n.1) Chromophobe adenoma (n.1)	Brain edema was shown in all cases. The microscopic study diagnosed the type-specific tumor.	Death was attributed in all cases to hydrocephalus and intracranial hypertension.
Lau, G. [24]	Case Report	Intracranial Germinoma	Macroscopic examination was unremarkable with a normal brain size and weight. At microscopic examination the pituitary gland displayed a massive tumor invasion with extensive peripheral lymphoid aggregates.	Death was due to a combination of acute hemorrhage of the tumor combined with microvascular disease of the heart.
Shiferaw, K. [25]	Case Report	Glioblastoma multiform	Macroscopic findings of the brain showed a tumor occupying both frontal lobes with extensive hemorrhagic infiltration and necrosis confirmed also on histological examination.	Death was attributed in all cases to hydrocephalus and intracranial hypertension.
Matturri, L. [26]	Case Report	Hemangioendothelioma	Macroscopic and microscopic examination of the brain found a solid tumor inside the medulla oblongata.	Death was due to the impaired breathing control due to the location of the tumor.
Matsumoto, H. [27]	Case Report	Glioblastoma multiform	Macroscopic findings of the brain were characterized by diffuse hypoxia/ischemia and edema of the brain tissue with extensive hemorrhagic infiltration and necrosis confirmed also on histological examination.	Death was attributed in all cases to hydrocephalus and intracranial hypertension.
Prahlow, J.A. [28]	Case Report	Astrocytoma	Macroscopic examination of the brain showed a solid tumor of 1.2 cm. Microscopically, cells of various shapes with nuclei of different size, and microcalcifications of the parenchyma were shown.	Death was due to a seizure disorder related to the tumor combined with acute ethanol intoxication.
Shields, L.B. [29]	Case Report	Pituitary adenoma	The pituitary fossa showed the presence of a red-colored tumor with hemorrhagic infarction. The examination in H&E revealed the presence of tumor with hemorrhagic infiltration.	Death was attributed to intracranial hypertension.
Ortiz-Reyes, R. [30]	Case Report	Subependymoma	Gross examination of the brain showed bilateral ventricular dilatation and inside a tumor of 3 cm in diameter. Microscopic examination showed meningothelial tumoral cells.	Death was due to the impaired breathing control due to the location of the tumor.
Nelson, J. [31]	Case Report	Ganglioma of the medulla	Examination of the brain after fixation showed a medulla with a mass invading the cerebellum. Microscopic examination revealed two types of neoplastic cells: astrocytes and oligodendrocytes.	Death was attributed to intracranial hypertension.

## Data Availability

All data are included in the main text.
